# Supplementary material for: Getting More Out of Biomedical Documents with GATE's Full Lifecycle Open Source Text Analytics
Source: PLoS Comput Biol. 2013 Feb 7;9(2):e1002854. doi: 10.1371/journal.pcbi.1002854 (PMC3567135; doi:10.1371/journal.pcbi.1002854)
Supplement: Dataset S1 — GATE software. Dataset S1 bundled with this paper contains a distribution of GATE (or see http://gate.ac.uk/download/). (TGZ) [file pcbi.1002854.s001.tgz › plos-gate/doc/papers.html]

GATE papers


# GATE papers

## Which paper to cite if ..

### I used GATE:

- H. Cunningham, et al. Text Processing with GATE (Version 6). University of Sheffield Department of Computer Science. 15 April 2011. ISBN 0956599311. Available from Amazon. BibTex.

### I Used ANNIE:

- H. Cunningham, D. Maynard, K. Bontcheva, V. Tablan. GATE: A Framework and Graphical Development Environment for Robust NLP Tools and Applications. Proceedings of the 40th Anniversary Meeting of the Association for Computational Linguistics (ACL'02). Philadelphia, July 2002. PDF. BibTeX.

### I used the ontology features in GATE:

- K. Bontcheva, V. Tablan, D. Maynard, H. Cunningham. Evolving GATE to Meet New Challenges in Language Engineering. Natural Language Engineering. 10 (3/4), pp. 349-373. 2004. Pre-print. BibTeX.

### I used GATE Mímir:

- H. Cunningham, V. Tablan, I. Roberts, M. A. Greenwood, and N. Aswani. Information Extraction and Semantic Annotation for Multi-Paradigm Information Management. In M. Lupu, K. Mayer, J. Tait, and A. J. Trippe, editors, Current Challenges in Patent Information Retrieval, volume 29 of The Information Retrieval Series. Springer, 2011. BibTeX.

### I used GATE Teamware:

- K. Bontcheva, H. Cunningham, I. Roberts, V. Tablan. Web-based Collaborative Corpus Annotation: Requirements and a Framework Implementation. In "New Challenges for NLP Frameworks" workshop at LREC, Malta, May 2010.PDF

### I used GATE for Information Retrieval

- H. Cunningham, A. Hanbury, and S. Rüger. Scaling up high-value retrieval to medium-volume data. In H. Cunningham, A. Hanbury, and S. Rüger, editors, Advances in Multidisciplinary Retrieval (the 1st Information Retrieval Facility Conference). LNCS volume number: 6107, Lecture Notes in Computer Science, Vienna, Austria, May 2010. Springer. BibTeX.

### On GATE and multimedia:

- M. Dowman, V. Tablan, H. Cunningham and B. Popov. Web-Assisted Annotation, Semantic Indexing and Search of Television and Radio News. 14th International World Wide Web Conference. Chiba, Japan, 2005. PDF. BibTeX.

### I used the machine learning facilities in GATE:

- Y. Li, K. Bontcheva and H. Cunningham. Adapting SVM for Data Sparseness and Imbalance: A Case Study on Information Extraction. Natural Language Engineering, 15(02), 241-271, 2009. On-line Link. BibTeX.

## Contents

- 1. Journals and Conference papers
- 2. PhD and MSc Theses
- 3. Tech reports etc.
- 4. Our BibTeX file.

# 1. Journals and Conference papers

## 2012

- D. Maynard and M. Greenwood. Large Scale Semantic Annotation, Indexing and Search at The National Archives. In Proceedings of LREC 2012, May 2012, Istanbul, Turkey. PDF

## 2011

- T. Risse, S. Dietze, D. Maynard and N. Tahmasebi. Using Events for Content Appraisal and Selection in Web Archives. In Proceedings of DeRiVE 2011: Workshop in conjunction with the 10th International Semantic Web Conference 2011, October 2011, Bonn, Germany. PDF

- D. Damljanovic, U. Kruschwitz and M. Albakour. Using Virtual Documents to move Information Retrieval and Knowledge Management Closer Together, 4th Workshop on Exploiting Semantic Annotation in Information Retrieval, collocated with the 20th ACM Conference on Information and Knowledge Management (CIKM) 2011, October 28, 2011

- V. Tablan, I. Roberts, H. Cunningham, and K. Bontcheva. GATECloud.net: Cloud Infrastructure for Large-Scale, Open-Source Text Processing. In UK e-Science All hands Meeting 2011, York, September 2011.PDF

- D. Maynard and A. Funk. Automatic detection of political opinions in tweets. In Raúl García-Castro, Dieter Fensel and Grigoris Antoniou (eds.) The Semantic Web: ESWC 2011 Selected Workshop Papers, Lecture Notes in Computer Science, Springer, 2011. PDF (longer version of MSM paper).

- D. Maynard and A. Funk. Automatic detection of political opinions in tweets. In Proceedings of MSM 2011: Making Sense of Microposts. Workshop at 8th Extended Semantic Web Conference (ESWC 2011). Heraklion, Greece. June 2011. PDF

- H. Wang, D. Damljanovic, T. Payne, N. Gibbins, and K. Bontcheva: Transition of Legacy Systems to Semantically Enabled Applications: TAO Method and Tools. Semantic Web Journal, IOS Press, 2011. (to appear)

- D. Damljanovic, M. Agatonovic, H. Cunningham: FREyA: an Interactive Way of Querying Linked Data using Natural Language. In Raúl García-Castro, Dieter Fensel and Grigoris Antoniou (eds.) The Semantic Web: ESWC 2011 Selected Workshop Papers, Lecture Notes in Computer Science, Springer, 2011. PDF (improved version of QALD-1 paper)

- D. Damljanovic, M. Agatonovic, H. Cunningham: FREyA: an Interactive Way of Querying Linked Data using Natural Language. In: Proceedings of 1st Workshop on Question Answering over Linked Data (QALD-1),
  Collocated with the 8th Extended Semantic Web Conference (ESWC 2011). Heraklion, Greece (June 2011) PDF

- D. Damljanovic, J. Petrak, M. Lupu, H. Cunningham, M. Carlsson, G. Engstrom, B. Andersson: Random Indexing for Finding Similar Nodes within Large RDF graphs. In Raúl García-Castro, Dieter Fensel and Grigoris Antoniou (eds.) The Semantic Web: ESWC 2011 Selected Workshop Papers, Lecture Notes in Computer Science, Springer, 2011. PDF (improved version of RED2011 paper)

- D. Damljanovic, J. Petrak, M. Lupu, H. Cunningham, M. Carlsson, G. Engstrom, B. Andersson: Random Indexing for Finding Similar Nodes within Large RDF graphs. In: Proceedings of the Fourth International Workshop on Resource Discovery (RED2011),
  Collocated with the 8th Extended Semantic Web Conference (ESWC 2011). Heraklion, Greece (June 2011) PDF

- M. Assel, A. Cheptsov, B. Czink, D. Damljanovic, J. Quesada: MPI Realization of High Performance Search for Querying Large RDF Graphs using Statistical Semantics . In: Proceedings of the 1st Workshop on High-Performance Computing for the Semantic Web, Collocated with the 8th Extended Semantic Web Conference (ESWC 2011). Heraklion, Greece (June 2011) PDF

## 2010

- H. Wang, D. Damljanovic, J. Sun. Enhanced Semantic Access to Formal Software Models. In the Proceedings of the 12th International Conference on Formal Engineering Methods, November 16 - 19, 2010, Shanghai, China. PDF

- K. Bontcheva, H. Cunningham, I. Roberts, V. Tablan. Web-based Collaborative Corpus Annotation: Requirements and a Framework Implementation. In "New Challenges for NLP Frameworks" workshop at LREC, Malta, May 2010.PDF

- Y. Zeng, Y. Wang, Z. Huang, D. Damljanovic, N. Zhong, and C. Wang. User Interests: Definition, Vocabulary, and Utilization in Unifying Search and Reasoning. Proceedings of the 2010 International Conference on Active Media Technology, Lecture Notes in Computer Science 6335, 98-107, 2010.

- D. Damljanovic: Towards Portable Controlled Natural Languages for Querying Ontologies. In: Rosner, M., Fuchs, N. (eds.) Second Workshop on Controlled Natural Languages, CEUR Workshop Pre-Proceedings ISSN 1613-0073, vol. 622. http://ceur-ws.org, Marettimo Island, Italy, 13-15 September, 2010. PDF

- A. Wyner, K. Angelov, G. Barzdins, D. Damljanovic, B. Davis, N. Fuchs, S. Hoefler, K. Jones, K. Kaljurand, T. Kuhn, M. Luts, J. Pool, M. Rosner, R. Schwitter, J. Sowa: On controlled natural languages: Properties and prospects. In N. Fuchs, ed.: Controlled Natural Language. Volume 5972 of Lecture Notes in Computer Science. Springer Berlin/Heidelberg (2010) 281–289 10.1007/978-3-642-14418-9 17.

- D. Damljanovic, J. Petrak, H. Cunningham: Random Indexing for Searching Large RDF Graphs. In the Proceedings of the 7th Extended Semantic Web Conference (ESWC 2010), Springer Verlag, Heraklion, Greece, May 31-June 3, 2010. Poster session, PDF

- D. Damljanovic, M. Agatonovic, H. Cunningham: Natural Language Interfaces to Ontologies: Combining Syntactic Analysis and Ontology-based Lookup through the User Interaction. In Proceedings of the 7th Extended Semantic Web Conference (ESWC 2010), Springer Verlag, Heraklion, Greece, May 31-June 3, 2010. PDF

- D. Damljanovic, M. Agatonovic, H. Cunningham: Identification of the Question Focus: Combining Syntactic Analysis and Ontology-based Lookup through the User Interaction. In Proceedings of the 7th Language Resources and Evaluation Conference (LREC 2010), ELRA 2010, La Valletta, Malta, May 17-23, 2010. PDF

- N. Aswani, R. Gaizauskas: Developing Morphological Analysers for South Asian Languages: Experimenting with the Hindi and Gujarati Languages. In Proceedings of the 7th Language Resources and Evaluation Conference (LREC 2010), ELRA 2010, La Valletta, Malta, May 17-23, 2010.

- N. Aswani, R. Gaizauskas: English-Hindi Transliteration using Multiple Similarity Metrics. In Proceedings of the 7th Language Resources and Evaluation Conference (LREC 2010), ELRA 2010, La Valletta, Malta, May 17-23, 2010.

- A. Funk, K. Bontcheva, Effective Development with GATE and Reusable Code for Semantically Analysing Heterogeneous Documents. In "New Challenges for NLP Frameworks" workshop at LREC, Malta, May 2010. PDF paper, slides

- A. Funk, I. Roberts, W. Peters, Implementing a Variety of Linguistic Annotations through a Common Web-Service Interface. In "Language Resource and Language Technology Standards" workshop at LREC, Malta, May 2010. PDF paper, slides

- A. Funk and K. Bontcheva, Ontology-Based Categorization of Web Services with Machine Learning. In Proceedings of the 7th Language Resources and Evaluation Conference (LREC 2010), ELRA 2010, La Valletta, Malta, May 17-23, 2010. PDF paper, poster

## 2009

- N. Aswani, R. Gaizauskas: Evolving a General Framework for Text Alignment: Case Studies with Two South Asian Languages. In Proceedings of the International Conference on Machine Translation: Twenty-Five Years On, Cranfield, Bedfordshire, UK, November 21-22, 2009.

- Agnoloni, T. Bacci, L. Francesconi, E. Peters, W. Montemagni, M., Venturi, G. (2009), A two-level knowledge approach to support multilingual legislative drafting. In: Breuker, J., Casanovas, P., Klein, M. and Francesconi, E. (eds.) Frontiers in Artificial Intelligence and Applications, Volume 188: Law, Ontologies and the Semantic Web - Channelling the Legal Information Flood

- F. Amardeilh and D. Damljanovic and K. Bontcheva: CA Manager: a Framework for Creating Customised Workflows for Ontology Population and Semantic Annotation. In the Proceedings of the Semantic Authoring, Annotation and Knowledge Markup Workshop (SAAKM 2009) co-located with the 5th International Conference on Knowledge Capture (K-CAP 2009), Redondo Beach, California, USA, 1-4 September, 2009. PDF

- F.Amardeilh and D.Damljanovic: Du texte a la connaissance : annotation semantique et peuplement d'ontologie appliques a des artefacts logiciels, Conference Ingenierie des Connaissances 2009, 25-29 May, 2009. (in French). PDF

- K. Bontcheva, B. Davis, A. Funk, Y. Li and T. Wang. Human Language Technologies. Semantic Knowledge Management, John Davies, Marko Grobelnik, and Dunja Mladenic (Eds.), Springer, 37-49, 2009. On-line Link

- D. Damljanovic, F. Amardeilh, K. Bontcheva: CA Manager Framework: Creating Customised Workflows for Ontology Population and Semantic Annotation. In Proceedings of The Fifth International Conference on Knowledge Capture (KCAP'09), California, USA, September 1-4, 2009.PDF.

- D. Damljanovic, K. Bontcheva: Towards Enhanced Usability of Natural Language Interfaces to Knowledge Bases. In Devedzic V. and Gasevic D. (Eds.), Special issue on Semantic Web and Web 2.0, Annals of Information systems, Springer-Verlag, 2009. PDF

- D. Damljanovic, M. Agatonovic, H. Cunningham: Usability of Natural Language Interfaces for querying ontologies presented at Workshop on Controlled Natural Language (CNL 2009 ), Marettimo Island, Italy, June 08-10, 2009.

- B.Davis, P.Varma, S.Handschuh, L.Dragan, H.Cunningham: Controlled Natural Language for Semantic Annotation. In Workshop on Controlled Natural Language (CNL) 2009, Marettimo Island, Italy, 2009.

- B.Davis, P.Varma, S.Handschuh, L.Dragan, H.Cunningham: On designing Controlled Natural Languages for Semantic Annotation. In Proceedings of the Workshop on Controlled Natural Language (CNL09), Springer-Verlag, Heidelberg, Germany. (In Press) Marettimo Island, Italy, 2009.

- M. Laclavik and D. Maynard. Motivating intelligent email in business: an investigation into current trends for email processing and communication research. Proceedings of Workshop on Emails in e-Commerce and Enterprise Context, 11th IEEE Conference on Commerce and Enterprise Computing, Vienna, Austria, 2009. PDF

- Y. Li, K. Bontcheva and H. Cunningham. Adapting SVM for Data Sparseness and Imbalance: A Case Study on Information Extraction. Natural Language Engineering, 15(02), 241-271, 2009. On-line Link

- D. Maynard and A. Funk and W. Peters. Using Lexico-Syntactic Ontology Design Patterns for ontology creation and population. In Proceedings of ISWC Workshop on Ontology Patterns (WOP 2009), Washington, 2009. PDF

- D. Maynard and A. Funk and W. Peters. NLP-based support for ontology lifecycle development. Proceedings of ISWC Workshop on Collaborative Construction, Management and Linking of Ontologies (CK 2009), Washington, 2009. PDF

- D. Maynard and A. Funk and W. Peters. SPRAT: a tool for automatic semantic pattern-based ontology population. Proceedings of the International Conference for Digital Libraries and the Semantic Web, Trento, Italy, 2009. PDF

- W.Peters: Text-based Legal Ontology Enrichment, In: proceedings of LOAIT 2009, Barcelona, Spain.PDF

- H.Saggion, A.Funk: Extracting Opinions and Facts for Business Intelligence. Revue des Nouvelles Technologies de l’Information (RNTI), no. E-17 pp119-146; November 2009. PDF

## 2008

- M. Agatonovic, N. Aswani, K. Bontcheva, H. Cunningham, T. Heitz, Y. Li, I. Roberts and V. Tablan. Large-scale, Parallel Automatic Patent Annotation. In Proceedings of 1st International CIKM Workshop on Patent Information Retrieval - PaIR'08, Napa Valley, California, USA, 30 October, 2008. PDF

- D.Damljanovic, V.Tablan, K.Bontcheva: A text-based query interface to owl ontologies. In: 6th Language Resources and Evaluation Conference (LREC), Marrakech, Morocco, ELRA (May 2008). PDF

- Damljanovic, D.,Bontcheva, K.. Enhanced Semantic Access to Software Artefacts. In Workshop on Semantic Web Enabled Software Engineering (SWESE) held in conjunction with ISWC'08, Karlsruhe, Germany, October 2008. PDF

- Brian Davis, Ahmad Ali Iqbal, Adam Funk, Valentin Tablan, Kalina Bontcheva, Hamish Cunningham and Siegfried Handschuh: RoundTrip Ontology Authoring. In *Proceedings of the 7th International Semantic Web Conference (ISWC2008)*, Karlsruhe, Germany, October 2008.

- E. Della Valle, D. Cerizza, I. Celino, A. Turati, H. Lausen, N. Steinmetz, M. Erdmann and A. Funk. Service-Finder: realizing Web Service Discovery at Web Scale. In Proceedings of European Semantic Technology Conference (ESTC 2008), Vienna, Austria, September, 2008. Video

- Adam Funk, Yaoyong Li, Horacio Saggion, Kalina Bontcheva and Christian Leibold: Opinion Analysis for Business Intelligence Applications. In *First International Workshop on Ontology-supported Business Intelligence (OBI2008)* at the *7th International Semantic Web Conference (ISWC)*, Karlsruhe, Germany, October 2008. PDF

- Y. Li and K. Bontcheva . Adapting Support Vector Machines for F-term-based Classification of Patents. ACM Transactions on Asian Language Information Processing, 7(2), 7:1--7:19, 2008. Link

- Y. Li and H. Cunningham. Geometric and Quantum Methods for Information Retrieval. SIGIR Forum, 42(2), 22-32, 2008. Link

- D. Maynard. Benchmarking Textual Annotation Tools for the Semantic Web. In Proc. of 6th International Conference on Language Resources and Evaluation (LREC), Marrakech, Morocco, 2008. PDF

- D. Maynard, Y. Li and W. Peters. NLP Techniques for Term Extraction and Ontology Population. Bridging the Gap between Text and Knowledge - Selected Contributions to Ontology Learning and Population from Text, P. Buitelaar and P. Cimiano (editors). IOS Press, 2007. PDF

- D. Maynard, W. Peters and Y. Li. Evaluating Evaluation Metrics for Ontology-Based Applications: Infinite Reflection. In Proc. of 6th International Conference on Language Resources and Evaluation (LREC), Marrakech, Morocco, 2008. PDF

- Montiel-Ponsoda, E., Aguado de Cea, G. and Gomez-Perez, A., and Peters, W., Modeling multilinguality in ontologies, In: Proceedings of Coling 2008, Manchester, U.K.PDF

- V. Tablan, D. Damljanovic, and K. Bontcheva. A natural language query interface to structured information. In Proceedings of the 5h European Semantic Web Conference (ESWC 2008), Tenerife, Spain, June, 2008. PDF

## 2007

- H. Saggion, A. Funk, D. Maynard, K. Bontcheva. Ontology-based Information Extraction for Business Applications. In Proceedings of the 6th International Semantic Web Conference (ISWC 2007), Busan, Korea, November, 2007. PDF

- A. Funk, V. Tablan, K. Bontcheva, H. Cunningham, B. Davis, S. Handschuh. CLOnE: Controlled Language for Ontology Editing. In Proceedings of the International Semantic Web Conference (ISWC 2007), Busan, Korea, November, 2007. PDF

- A. Funk, D. Maynard, H. Saggion and K. Bontcheva. Ontological Integration of Information Extracted from Multiple Sources. In the Multi-source Multilingual Information Extraction and Summarization (MMIES) workshop at Recent Advances in Natural Language Processing (RANLP07), Borovets, Bulgaria, September, 2007. PDF

- Y. Li, K. Bontcheva and H. Cunningham. Hierarchical, Perceptron-like Learning for Ontology Based Information Extraction. Proceedings of the 16th International World Wide Web Conference (WWW2007), pp. 777-786. May, 2007. PDF

- Y. Li, K. Bontcheva and H. Cunningham. Cost Sensitive Evaluation Measures for F-term Patent Classification. Proceedings of the First International Workshop on Evaluating Information Access (EVIA 2007), pp. pp. 44-53. May, 2007. PDF

- Y. Li, K. Bontcheva and H. Cunningham. SVM Based Learning System for F-term Patent Classification. Proceedings of the Sixth NTCIR Workshop Meeting on Evaluation of Information Access Technologies: Information Retrieval, Question Answering and Cross-Lingual Information Access, pp. 396-402. May, 2007. PDF

- Y. Li, K. Bontcheva and H. Cunningham. Experiments of Opinion Analysis on the Corpora MPQA and NTCIR-6. Proceedings of the Sixth NTCIR Workshop Meeting on Evaluation of Information Access Technologies: Information Retrieval, Question Answering and Cross-Lingual Information Access, pp. 323-329. May, 2007. PDF

- Y. Li and J. Shawe-Taylor. Advanced Learning Algorithms for Cross-language Patent Retrieval and Classification. Information Processing and Management, 43(5), pp. 1183-1199. 2007. Link

- D. Maynard, H. Saggion, M. Yankova, K. Bontcheva and W. Peters. Natural Language Technology for Information Integration in Business Intelligence. 10th International Conference on Business Information Systems, Poland, 25-27 April, 2007. PDF

- D. Maynard, W. Peters, M. d'Aquin and M. Sabou. Change Management for Metadata Evolution. In Proceedings of ESWC International Workshop on Ontology Dynamics (IWOD), Innsbruck, Austria, June, 2007. PDF

- Peters, W., Sagri, M., and Tiscornia, D. 2007. The structuring of legal knowledge in LOIS. Artfiicial Intelligence and Law, 15, 2 (Jun. 2007), 117-135.Link

- Peters, W., Montiel-Ponsoda, E., Aguado de Cea, G. and Gomez-Perez, A., Localizing Ontologies in Owl, ISWC 2007 workshop Ontolex2007, Busan, Korea PDF

- Montiel-Ponsoda, E. Aguado de Cea, G. Suarez-Figueroa, M.C. Palma, R. Peters, W., LexOMV: An OMV extension to capture multilinguality. ISWC 2007 workshop Ontolex 2007, Busan, Korea PDF

- H.Saggion, SHEF: Semantic Tagging and Summarization Techniques Applied to Cross-document Coreference. In Proceedings of Semeval 2007, Association for Computational Linguistics, June, 2007. PDF

## 2006

- N.Aswani, K.Bontcheva ;and H.Cunningham: ;Mining Information for Instance Unification. In the proceedings of 5th International Semantic Web conference, Athens, GA, USA, November, 2006. PDF

- K. Bontcheva, M. Sabou. Learning Ontologies from Software Artifacts: Exploring and Combining Multiple Sources. In proceedings of Workshop on Semantic Web Enabled Software Engineering (SWESE), November, 2006. PDF

- K. Bontcheva, J. Davies, A. Duke, T. Glover, N. Kings, I. Thurlow. Semantic Information Access. In Semantic Web Technologies: Trends and Research in Ontology-based Systems. J. Davies, R. Studer, P. Warren (eds). John Wiley, 2006. Link

- K.Bontcheva, H.Cunningham, A.Kiryakov and V.Tablan. Semantic Annotation and Human Language Technology. Semantic Web Technology: Trends and Research. John Wiley and Sons Ltd. 2006. Abstract Link

- B. Davis, Siegfried Handschuh, H. Cunningham and V. Tablan, Further use of Controlled Natural Language for Semantic Annotation of Wikis. In the proceedings of the 1st Semantic Authoring and Annotation Workshop at ISWC2006, Athens, GA, USA, November, 2006. PDF

- Y. Li, J. Shawe-Taylor. Using KCCA for Japanese-English Cross-language Information Retrieval and Document Classification. Journal of Intelligent Information Systems, 27(2), 117-133. 2006. Link

- D. Maynard, W. Peters, Y. Li. Metrics for Evaluation of Ontology-based Information Extraction. In Proc of WWW 2006 Workshop on ";Evaluation of Ontologies for the Web"; (EON 2006), Edinburgh, Scotland, 2006. PDF

- V.Tablan, T.Polajnar, ;H.Cunningham,K.Bontcheva. User-friendly ontology authoring using a controlled language. Proceedings of the 5th International Conference on Language Resources and Evaluation (LREC), Genoa, Italy, May, 2006. PDF

- T. Wang, Y. Li, K. Bontcheva, H. Cunningham, J. Wang. Automatic Extraction of Hierarchical Relations from Text. Proceedings of the Third European Semantic Web Conference (ESWC 2006), Lecture Notes in Computer Science 4011, Springer, 2006. PDF

## 2005

- N. Aswani, V. Tablan, K. Bontcheva and H. Cunningham. Indexing and Querying Linguistic Metadata and Document Content. Proceedings of Fifth International Conference on Recent Advances in Natural Language Processing (RANLP-2005), 2005. PDF

- K. Bontcheva. Generating Tailored Textual Summaries from Ontologies. Second European Semantic Web Conference (ESWC'05), Crete, 2005. PDF

- G. Crane, K. Bontcheva, J. A. Rydberg-Cox, C. E. Wulfman. Emerging language technologies and the rediscovery of the past: a research agenda. International Journal on Digital Libraries 5(4): 309-316. 2005. Link

- H. Cunningham. Information Extraction, Automatic. Encyclopedia of Language and Linguistics, 2nd Edition, Elsevier. 2005. preprint PDF

- H. Cunningham, K. Bontcheva. Computational Language Systems, Architectures. Encyclopedia of Language and Linguistics, 2nd Edition, Elsevier. 2005. preprint PDF

- H. Cunningham, K. Bontcheva, Y. Li. Knowledge Management and Human Language: Crossing the Chasm. Journal of Knowledge Management, Vol. 9, No. 5. pp. 108--131. 2005. Abstract

- J. Davies, A. Duke, N. Kings, D. Mladenic, K. Bontcheva, M. Grcar, R. Benjamins, J. Contreras, M. B. Civico, T. Glover. Next Generation Knowledge Access. Journal of Knowledge Management, Vol. 9, No. 5. pp. 64--84. 2005. Abstract

- M. Dowman, V. Tablan, H. Cunningham and B. Popov. Web-Assisted Annotation, Semantic Indexing and Search of Television and Radio News. 14th International World Wide Web Conference. Chiba, Japan, 2005. PDF

- M. Dowman, V. Tablan, H. Cunningham and B. Popov. Content Augmentation for Mixed-Mode News Broadcasts. 3rd European Conference on Interactive Television: User Centred ITV Systems, Programmes and Applications. Aalborg University, Denmark, 2005. PDF.

- M. Dowman, V. Tablan, H. Cunningham, C. Ursu and B. Popov. Semantically Enhanced Television News through Web and Video Integration. Second European Semantic Web Conference (ESWC'05), Crete, 2005. PDF

- Y. Li and C. Miao and K. Bontcheva and H. Cunningham. Perceptron Learning for Chinese Word Segmentation. Proceedings of Fourth SIGHAN Workshop on Chinese Language processing (Sighan-05), pp. 154-157. 2005. PDF

- Y. Li, K. Bontcheva, and H. Cunningham. Using Uneven Margins SVM and Perceptron for Information Extraction. Proceedings of Ninth Conference on Computational Natural Language Learning (CoNLL-2005), pp. 72-79. 2005. PDF

- Y. Li, K. Bontcheva, and H. Cunningham. SVM Based Learning System For Information Extraction. J. Winkler, M. Niranjan and N. Lawerence (Eds.): Deterministic and Statistical Methods in Machine Learning, LNAI 3635, Springer Verlag, pp. 319-339. 2005. PDF

- D. Maynard. Benchmarking ontology-based annotation tools for the Semantic Web. UK e-Science Programme All Hands Meeting (AHM2005) Workshop ";Text Mining, e-Research and Grid-enabled Language Technology";, Nottingham, UK, 2005. PDF

- D. Maynard, M. Yankova, A. Kourakis and A. Kokossis. Ontology-based information extraction for market monitoring and technology watch, ESWC Workshop ";End User Aspects of the Semantic Web";, Heraklion, Crete, 2005. PDF

- C. Ursu, V. Tablan and H. Cunningham. Semantic Analysis for Tomorrow's Audio-Visual Digital Archives. Proceedings of 2nd European Workshop on the Integration of Knowledge, Semantic and Digital Media Technologies (EWIMT-2005), London, UK, 2005. PDF

## 2004

- K. Bontcheva. Open-source Tools for Creation, Maintenance, and Storage of Lexical Resources for Language Generation from Ontologies. Fourth International Conference on Language Resources and Evaluation (LREC'2004). Lisbon, Portugal. 2004. PDF.

- Bontcheva, V. Tablan, D. Maynard, H. Cunningham. Evolving GATE to Meet New Challenges in Language Engineering. Natural Language Engineering. 10 (3/4), pp. 349-373. 2004. PDF

- K. Bontcheva, Y. Wilks. Automatic Report Generation from Ontologies: the MIAKT approach. Nineth International Conference on Applications of Natural Language to Information Systems (NLDB'2004). Manchester, UK. 2004. PDF.

- H. Cunningham, D. Scott. Journal of Natural Language Engineering. Introduction to the Special Issue on Software Architecture for Language Engineering. 2004. PDF

- A. Dalli, V. Tablan, K. Bontcheva, Y. Wilks, D. Broeder, H. Brugman, P. Wittenburg. Web Services Architecture for Language Resources. Fourth International Conference on Language Resources and Evaluation (LREC'2004). Lisbon, Portugal. 2004.PS

- M. Dimitrov, K. Bontcheva, H. Cunningham, D. Maynard, A Light-weight Approach to Coreference Resolution for Named Entities, In Anaphora Processing: Linguistic, Cognitive and Computational Modelling, Antonio Branco, Tony McEnery and Ruslan Mitkov (editors), 2004. Link

- Y. Li and K. Bontcheva and H. Cunningham. An SVM Based Learning Algorithm for Information Extraction. Machine Learning Workshop. Sheffield, UK. 2004. PDF

- D. Maynard, K. Bontcheva, H. Cunningham. Automatic Language-Independent Induction of Gazetteer Lists. Fourth International Conference on Language Resources and Evaluation (LREC'2004). Lisbon, Portugal. 2004. PS

- D. Maynard, M. Yankova, N. Aswani, H. Cunningham. Automatic Creation and Monitoring of Semantic Metadata in a Dynamic Knowledge Portal. Proceedings of the 11th International Conference on Artificial Intelligence: Methodology, Systems, Applications (AIMSA 2004), Varna, Bulgaria, 2004. PDF

- M.M. Wood, S.J. Lydon, V. Tablan, D. Maynard, H. Cunningham. Populating a Database from Parallel Texts using Ontology-based Information Extraction. NLDB 2004, 2004. PDF

## 2003

- K. Bontcheva, H. Cunningham. The Semantic Web: A New Opportunity and Challenge for Human Language Technology. Workshop on Human Language Technology for the Semantic Web and Web Services. Held in conjunction with the Second International Semantic Web Conference (ISWC'03). H. Cunningham, Y. Ding, A. Kiryakov (eds). Florida, USA. October 2003. PDF

- K. Bontcheva, A. Kiryakov, H. Cunningham, B. Popov. M. Dimitrov. Semantic Web Enabled, Open Source Language Technology. Language Technology and the Semantic Web, Workshop on NLP and XML (NLPXML-2003), held in conjunction with EACL 2003, Budapest, 2003. PDF

- K. Bontcheva, D.Maynard, V. Tablan, and H. Cunningham. GATE: A Unicode-based Infrastructure Supporting Multilingual Information Extraction. Workshop on Information Extraction for Slavonic and other Central and Eastern European Languages. Held in conjunction with the 4th International Conference ";Recent Advances in Natural Language Processing"; (RANLP'2003), Bulgaria, 2003. PDF

- H. Cunningham, V. Tablan, K. Bontcheva, M. Dimitrov. Language engineering tools for collaborative corpus annotation. Proceedings of Corpus Linguistics 2003, Lancaster, UK, 2003. DOC

- D.Maynard. Multi-Source and Multilingual Information Extraction. Expert Update, 2003. PDF

- D.Maynard, K. Bontcheva and H. Cunningham. Towards a semantic extraction of named entities. Recent Advances in Natural Language Processing, Bulgaria, 2003. PDF

- D. Maynard, H. Cunningham. Multilingual adaptations of a reusable information extraction tool. EACL 2003, Budapest, 2003. PDF

- D. Maynard, V. Tablan, K. Bontcheva, H. Cunningham. Rapid customisation of an Information Extraction system for surprise languages. ACM Transactions on Asian Language Information Processing, Special issue on Rapid Development of Language Capabilities: The Surprise Languages. 2003. Link

- D.Maynard, V. Tablan and H. Cunningham. NE recognition without training data on a language you don't speak. ACL Workshop on Multilingual and Mixed-language Named Entity Recognition: Combining Statistical and Symbolic Models, Sapporo, Japan, 2003. PDF

- D. Manov and A. Kiryakov and B. Popov and K. Bontcheva and D. Maynard, H. Cunningham. Experiments with geographic knowledge for information extraction. Workshop on Analysis of Geographic References, HLT/NAACL'03, Edmonton, Canada, 2003. PDF

- H. Saggion, K. Bontcheva, H. Cunningham. Robust Generic and Query-based Summarisation. Proceedings of the European Chapter of Computational Linguistics (EACL), Research Notes and Demos, 2003. Link

- H. Saggion, H. Cunningham, K. Bontcheva, D. Maynard, O. Hamza, Y. Wilks. Multimedia Indexing through Multi-source and Multi-language Information Extraction: The MUMIS Project. Data & Knowledge Engineering Journal, Volume 48, pp. 247-264, 2003. Link

- H. Saggion, J. Kuper, T. Declerck, D. Reidsma, H. Cunningham. Intelligent Multimedia Indexing and Retrieval through Multi-source Information Extraction and Merging. IJCAI 2003, Acapulco, Mexico, 9-15 August 2003. PDF

- V. Tablan, K. Bontcheva, D. Maynard, H. Cunningham. OLLIE: On-Line Learning for Information Extraction. HLT-NAACL 2003 Workshop: Software Engineering and Architecture of Language Technology Systems (SEALTS). Edmonton, Canada, 2003. PDF

- I.Witten, K.Don, M.Dewsnip, V.Tablan. Text mining in a digital library. Journal of Digital Libraries special issue ";Digital Libraries as experienced by the editors of the Journal"; 2003. PDF

- M. M. Wood and S. J. Lydon and V. Tablan and D. Maynard and H. Cunningham. Using parallel texts to improve recall in IE. Recent Advances in Natural Language Processing, Bulgaria, 2003.

## 2002 and previous

- P. Baker and A. Hardie and T. McEnery and H. Cunningham and R. Gaizauskas. EMILLE, A 67-Million Word Corpus of Indic Languages: Data Collection, Mark-up and Harmonisation. Proceedings of 3rd Language Resources and Evaluation Conference (LREC 2002) pp. 819-825. PDF

- K. Bontcheva, H. Cunningham, V. Tablan, D.Maynard, O.Hamza. Using GATE as an Environment for Teaching NLP. Proceedings of the ACL Workshop on Effective Tools and Methodologies in Teaching NLP, 2002. PDF

- K. Bontcheva, H. Cunningham, V. Tablan, D. Maynard, H. Saggion. Developing Reusable and Robust Language Processing Components for Information Systems using GATE. 3rd International Workshop on Natural Language and Information Systems (NLIS'2002), IEEE Computer Society Press, 2002. PS

- K. Bontcheva, M. Dimitrov, D. Maynard, V. Tablan, H. Cunningham. Shallow Methods for Named Entity Coreference Resolution. Chaines de references et resolveurs d'anaphores, workshop TALN 2002, Nancy, France, 2002. PDF

- K. Bontcheva, D. Maynard, H. Cunningham, H. Saggion. Using Human Language Technology for Automatic Annotation and Indexing of Digital Library Content. Proceedings of the 6th European Conference on Research and Advanced Technology for Digital Libraries (ECDL'2002), Rome, September 2002. PDF

- H. Cunningham, K. Bontcheva, D. Maynard, V. Tablan. GATE -- A New Release. ELSNews, 11(1), 2002. PDF

- H. Cunningham, D. Maynard, K. Bontcheva, V. Tablan. GATE: A Framework and Graphical Development Environment for Robust NLP Tools and Applications. Proceedings of the 40th Anniversary Meeting of the Association for Computational Linguistics (ACL'02). Philadelphia, July 2002. PDF.

- H. Cunningham. GATE, a General Architecture for Text Engineering. Computers and the Humanities, volume 36, pp. 223-254, 2002. PDF

- M. Dimitrov, K. Bontcheva, H. Cunningham, D. Maynard. A Light-weight Approach to Coreference Resolution for Named Entities in Text. Proceedings of the Fourth Discourse Anaphora and Anaphor Resolution Colloquium (DAARC), Lisbon, September 2002. PDF

- P. Lal and S. Ruger. Extract-based Summarization with Simplification. Proceedings of the ACL Workshop on Text Summarisation / DUC 2002, Philadelphia, July 2002. PDF

- D.Maynard, K. Bontcheva, H. Saggion, H. Cunningham, O. Hamza. Using a Text Engineering Framework to Build an Extendable and Portable IE-based Summarisation System. Proceedings of the ACL Workshop on Text Summarisation, Philadelphia, July 2002. PDF

- D. Maynard, H. Cunningham, K. Bontcheva, M. Dimitrov. Adapting A Robust Multi-Genre NE System for Automatic Content Extraction. Proceedings of the Tenth International Conference on Artificial Intelligence: Methodology, Systems, Applications (AIMSA 2002), 2002. PDF.

- D. Maynard, H.Cunningham, R.Gaizauskas. Named Entity Recognition at Sheffield University. Nordic Language Technology -- Arbog for Nordisk Sprogtechnologisk Forskningsprogram 2002-2004, 141-145, Museum Tusculanums Forlag. Maynard, V. Tablan, H. Cunningham, C. Ursu, H. Saggion, K. Bontcheva and Y. Wilks. Architectural Elements of Language Engineering Robustness. Journal of Natural Language Engineering -- Special Issue on Robust Methods in Analysis of Natural Language Data, Volume 8, Number 2-3, 257-274, 2002. PDF

- K. Pastra, D. Maynard, H. Cunningham, O. Hamza, Y. Wilks. How feasible is the reuse of grammars for Named Entity Recognition? Language Resources and Evaluation Conference (LREC'2002), 2002. PS

- H. Saggion. Shallow-based Robust Summarization. Workshop on Text Summarization (ATALA), Paris, France, 2002.

- H. Saggion, H. Cunningham, K. Bontcheva, D. Maynard, C. Ursu, O. Hamza, Y. Wilks. Access to Multimedia Information through Multisource and Multilanguage Information Extraction. 7th Workshop on Applications of Natural Language to Information Systems (NLDB 2002), Stockholm, Sweden, 2002. Link

- H. Saggion, H. Cunningham, D. Maynard, K. Bontcheva, O. Hamza, C. Ursu, Y. Wilks. Extracting Information for Information Indexing of Multimedia Material. Language Resources and Evaluation Conference (LREC'2002), 2002. PS

- V. Tablan, C. Ursu, K. Bontcheva, H. Cunningham, D. Maynard, O. Hamza, T. McEnery, P. Baker, M. Leisher. A Unicode-based Environment for Creation and Use of Language Resources. Language Resources and Evaluation Conference (LREC'2002), 2002. PDF

- K. Bontcheva, C. Brewster, F. Ciravegna, H. Cunningham, L. Guthrie, R. Gaizauskas, Y. Wilks. Using HLT for Acquiring, Retrieving and Publishing Knowledge in AKT. 2001 EACL/ACL Workshop on Human Language Technology and Knowledge Management, Toulouse, France, July 2001. PDF

- D. Maynard and V. Tablan and C. Ursu and H. Cunningham and Y. Wilks. Named Entity Recognition from Diverse Text Types. Recent Advances in Natural Language Processing 2001 Conference, Tzigov Chark, Bulgaria, 2001.PDF

- Gamback, Bjorn and Olsson, Fredrik. Experiences of Language Engineering Algorithm Reuse. In Proceedings of the Second Conference on Language Resources Evaluation, Athens, 2000. PS

- K. Bontcheva and H. Brugman and A. Russel and P. Wittenburg and H. Cunningham. An Experiment in Unifying Audio-Visual and Textual Infrastructures for Language Processing R&D. In Workshop on Using Toolsets and Architectures To Build NLP Systems at COLING-2000, Luxembourg, 2000. PS

- H. Cunningham, K. Bontcheva, W. Peters, Y. Wilks. Uniform language resource access and distribution in the context of a General Architecture for Text Engineering (GATE). Proceedings of the Workshop on Ontologies and Language Resources (OntoLex'2000), Sozopol, Bulgaria, 8-10 September, 2000. PS

- H. Cunningham and K. Bontcheva and V. Tablan and Y. Wilks. Software Infrastructure for Language Resources: a Taxonomy of Previous Work and a Requirements Analysis. In Proceedings of the Second Conference on Language Resources Evaluation, Athens, 2000. Abstract. PDF. HTML

- H. Cunningham and D. Maynard and K. Bontcheva and V. Tablan and Y. Wilks. Experience of using GATE for NLP R&D. In Workshop on Using Toolsets and Architectures To Build NLP Systems at COLING-2000, Luxembourg, 2000. PostScript. HTML

- A.M. McEnery and P. Baker and R. Gaizauskas and H. Cunningham. EMILLE: Building a Corpus of South Asian Languages. Vivek, A Quarterly in Artificial Intelligence. Number 3, Volume 13, Pages 23-32. 2000. Link

- H. Cunningham and R. Gaizauskas, and K. Humphreys and Y. Wilks. Experience with a Language Engineering Architecture: Three Years of GATE. In *Proceedings of the AISB'99 Workshop on Reference Architectures and Data Standards for NLP*, The Society for the Study of Artificial Intelligence and Simulation of Behaviour, Edinburgh, U.K. Apr, 1999. HTML.

- H. Cunningham and W. Peters and C. McCauley and K. Bontcheva and Y. Wilks. A Level Playing Field for Language Resource Evaluation. In *Workshop on Distributing and Accessing Lexical Resources at Conference on Language Resources Evaluation, Granada, Spain*, 1998. HTML. PostScript.

- H. Cunningham and M. Stevenson and Y. Wilks. Implementing a Sense Tagger within a General Architecture for Language Engineering. Proceedings of the Third Conference on New Methods in Language Engineering (NeMLaP-3), pages 59-72, Sydney, Australia, 1998. PDF

- W. Peters and H. Cunningham and C. McCauley and K. Bontcheva and Y. Wilks. Uniform Language Resource Access and Distribution. In *Workshop on Distributing and Accessing Lexical Resources at Conference on Language Resources Evaluation, Granada, Spain*, 1998. PS

- M. Stevenson and H. Cunningham and Y. Wilks. Sense Tagging and Language Engineering. In *Proceedings of the 13th European Conference on Artificial Intelligence (ECAI-98)*, pages 185-189, Brighton, UK, 1998. PS

- H. Cunningham and K. Humphreys and R. Gaizauskas and Y. Wilks. Software Infrastructure for Natural Language Processing. In *Proceedings of the Fifth Conference on Applied Natural Language Processing (ANLP-97)*, Mar, 1997. http://xxx.lanl.gov/abs/cs.CL/9702005. Local PostScript.

- H. Cunningham and K. Humphreys and R. Gaizauskas and Y. Wilks. GATE -- a TIPSTER-based General Architecture for Text Engineering. In *Proceedings of the TIPSTER Text Program (Phase III) 6 Month Workshop*, DARPA, Morgan Kaufmann, California, May, 1997. PostScript.

- H. Cunningham and K. Humphreys and R. Gaizauskas and Y. Wilks. TIPSTER-Compatible Projects at Sheffield. In *Advances in Text Processing, TIPSTER Program Phase II*, DARPA, Morgan Kaufmann, California, 1996. PS

- H. Cunningham and Y. Wilks and R. Gaizauskas. GATE -- a General Architecture for Text Engineering. In *Proceedings of the 16th Conference on Computational Linguistics (COLING-96)*, Copenhagen, Aug, 1996. PostScript.

- H. Cunningham and Y. Wilks and R. Gaizauskas. New Methods, Current Trends and Software Infrastructure for NLP. In *Proceedings of the Conference on New Methods in Natural Language Processing (NeMLaP-2)*, Bilkent University, Turkey, Sep, 1996. http://xxx.lanl.gov/abs/cs.CL/9607025. Local PostScript.

- H. Cunningham and Y. Wilks and R. Gaizauskas. Software Infrastructure for Language Engineering. In *Proceedings of the AISB Workshop on Language Engineering for Document Analysis and Recognition*, Brighton, U.K. Apr, 1996. PDF

- R. Gaizauskas, H. Cunningham, Y. Wilks, P. Rodgers, and K. Humphreys. GATE: An Environment to Support Research and Development in Natural Language Engineering. In *Proceedings of the 8th IEEE International Conference on Tools with Artificial Intelligence*, Toulouse, France, 1996. Abstract, Postscript

- H. Cunningham and M. Freeman and W.J. Black. Software Reuse, Object-Oriented Frameworks and Natural Language Processing. In *New Methods in Language Processing (NeMLaP-1), September 1994*, (Re-published in book form 1997 by UCL Press), Manchester, 1994.

# 2. PhD and MSc Theses

- H. Cunningham. Software Architecture for Language Engineering. *PhD Thesis*, University of Sheffield, 2000.

- M. Dimitrov. A Light-weight Approach to Coreference Resolution for Named Entities in Text. *MSc Thesis*, University of Sofia, Bulgaria, 2002. PDF

- P. Lal. Text Summarization. *MSc Thesis* , Imperial College, London, 2002. PDF.

# 3. Tech reports etc.

- T. Polajnar and V. Tablan and H. Cunningham. User-Friendly Ontology Authoring Using a Controlled Language. Technical report CS-05-10, University of Sheffield, Department of Computer Science, 2005. PDF.

- D. Maynard and V. Tablan and K. Bontcheva and H. Cunningham and Y, Wilks. MUlti-Source Entity recognition -- an Information Extraction System for Diverse Text Types. Technical report CS--02--03, University of Sheffield, Department of Computer Science, 2003. PostScript.

- A. Kiryakov. Technical Report CS--03--03, Department of Computer Science, University of Sheffield. Ontology and Reasoning in MUMIS: Towards the Semantic Web. 2003. Word.

- H. Cunningham and D. Maynard and V. Tablan. JAPE: a Java Annotation Patterns Engine (Second Edition). Technical report CS--00--10, University of Sheffield, Department of Computer Science, 2000. PostScript.

- D. Maynard and H. Cunningham and K. Bontcheva and R. Catizone and G. Demetriou and R. Gaizauskas and O. Hamza and M. Hepple and P. Herring and B. Mitchell and M. Oakes and W. Peters and A. Setzer and M. Stevenson and V. Tablan and C. Ursu and Y. Wilks. A Survey of Uses of GATE. Technical report CS--00--06, University of Sheffield, Department of Computer Science, 2000. HTML.

- H. Brugman and K. Bontcheva and P. Wittenburg and H. Cunningham. Integrating Multimedia and Textual Software Architectures for Language Technology. Max-Planck Institute for Psycholinguistics, Nijmegen, Nethedlands, 1999.

- H. Cunningham. JAPE: a Java Annotation Patterns Engine. Department of Computer Science, University of Sheffield, May, 1999. PDF

- H. Cunningham and K. Humphreys and R. Gaizauskas and M. Stower. CREOLE Developer's Manual. Department of Computer Science, University of Sheffield, Department of Computer Science, University of Sheffield. 1996.

- R. Gaizauskas and P. Rodgers and H. Cunningham and K. Humphreys. GATE User Guide. Department of Computer Science, University of Sheffield, Department of Computer Science, University of Sheffield. 1996.

- K. Humphreys and R. Gaizauskas and H. Cunningham and S. Azzam. CREOLE Module Specifications. Department of Computer Science, University of Sheffield, Department of Computer Science, University of Sheffield, 1996.

- H. Cunningham and R. Gaizauskas and Y. Wilks. A General Architecture for Text Engineering (GATE) -- a new approach to Language Engineering R&D. Department of Computer Science, University of Sheffield, 1995. http://xxx.lanl.gov/abs/cs.CL/9601009.

# 4. Our BibTeX file.

Download here.
